# Adrenocorticotropic Hormone and Thyroxine Concentrations in Young Healthy Equids: Species Differences and Seasonal Variations

**DOI:** 10.3390/ani16071124

**Published:** 2026-04-07

**Authors:** Natalia Siwińska, Agnieszka Żak-Bochenek, Paulina Jawor, Aleksandra Pietrzak, Oliwia Kutermak, Malwina Słowikowska-Łoś, Beata Kaczmarek

**Affiliations:** 1 Department of Internal Medicine and Clinic of Diseases of Horses, Dogs and Cats, Wrocław University of Environmental and Life Sciences, Grunwaldzki Sq. 47, 50-366 Wrocław, Poland; malwina.slowikowska-los@upwr.edu.pl; 2 Department of Immunology, Pathophysiology and Veterinary Preventive Medicine, Wrocław University of Environmental and Life Sciences, 31 Norwida St., 50-375 Wrocław, Poland; agnieszka.zak-bochenek@upwr.edu.pl (A.Ż.-B.); paulina.jawor@upwr.edu.pl (P.J.); 3 Faculty of Veterinary Medicine, Wrocław University of Environmental and Life Sciences, 31 Norwida St., 50-375 Wrocław, Poland; 122210@student.upwr.edu.pl (A.P.); 120260@student.upwr.edu.pl (O.K.); 4 Department and Clinic of Animal Internal Diseases, University of Life Sciences in Lublin, 15 Akademicka Str., 20-950 Lublin, Poland; beata.kaczmarek@up.lublin.pl

**Keywords:** donkeys, ponies, horses, ACTH, thyroxine

## Abstract

Due to growing interest in endocrine diseases, more studies aim to interpret the baseline concentrations and reference intervals of hormone values like adrenocorticotropic hormone (ACTH) and thyroxine (T4) in horses. Most studies available in the literature focus on evaluating ACTH and thyroid hormone values in adult and geriatric horses, with only a few considering younger donkeys and ponies. This study aimed to measure ACTH and T4 in autumn and spring in younger equids. The study included 42 young equids, which were divided into three groups: horses, ponies, and donkeys. The concentration of T4 was significantly higher in autumn than in spring in all the animals (*p* = 0.049) and significantly higher in donkeys than in horses (*p* = 0.0046). The concentration of ACTH was significantly higher in autumn than in spring in all animals (*p* = 0.00015) and significantly higher in donkeys than in horses and ponies (*p* = 0.001). The study results underscore the importance of utilizing seasonal hormone reference ranges specifically adapted for ponies and donkeys to accurately assess hormone secretion patterns in young animals. Reliance on reference values established for horses may lead to overinterpretation of hormone concentrations and misdiagnosis of endocrine disease in healthy donkeys or ponies.

## Simple Summary

Hormones such as adrenocorticotropic hormone (ACTH) and thyroxine (T4) are commonly measured in equids to support the diagnosis of endocrine disorders. Most available reference values are based on adult horses, while much less is known about normal hormone concentrations in young ponies and donkeys. In this study, ACTH and T4 concentrations were measured in young horses, ponies, and donkeys during autumn and spring. Hormone concentrations varied between seasons and differed among equine species, with donkeys showing higher ACTH concentrations than both horses and ponies, as well as higher T4 concentrations than horses. These findings highlight the importance of using species- and season-specific reference values when interpreting hormone test results in young equids. Applying reference ranges developed for horses to ponies or donkeys may lead to incorrect clinical interpretation in otherwise healthy animals.

## 1. Introduction

Endocrine regulation plays a crucial role in maintaining physiological homeostasis in animals and has been the subject of extensive research in equine medicine. Among the hormones of particular interest are adrenocorticotropic hormone (ACTH) and thyroid hormones, including thyroxine (T4), which are commonly evaluated in clinical and research settings. Assessment of their concentrations in young, clinically healthy equids is essential for improving the interpretation of endocrine test results and establishing appropriate physiological values. Under physiological conditions, ACTH production by the equine pituitary gland is known to undergo seasonal changes, with overproduction around the fall [1]. The seasonal variations in thyroid hormones remain incompletely understood; however, some months, such as March, are associated with increased T4 concentrations [1,2]. Alterations in the secretion of these hormones may occur in endocrine disorders such as pituitary pars intermedia dysfunction (PPID) in adult and geriatric animals, hypothyroidism, or hyperthyroidism. However, hormonal concentrations may also be influenced by factors unrelated to primary endocrine disease, including stress or concurrent systemic conditions [2,3]. Consequently, interpretation of endocrine test results can be challenging in certain situations, particularly in young animals with mildly increased hormone concentrations [4]. Most existing studies predominantly focus on evaluating ACTH and T4 in adult and senior horses, with limited research investigating these parameters in younger horses, as well as donkeys and ponies [5,6,7,8]. Reference values with diagnostic guidelines for endocrine testing in horses have been established for many years; however, only recent studies have addressed their applicability to donkeys and ponies. It is now known that donkeys of all ages can exhibit higher ACTH concentrations than horses, but they also show seasonal changes [5,6]. This species is also characterized by increased total serum T4 [7]. Assessment of ACTH concentrations in ponies has only been initiated in recent years, and current research remains limited. Studies have demonstrated that ACTH concentrations are significantly higher in Shetland and Welsh ponies compared to horses, particularly during autumn [8,9]. However, there is no data focused on the evaluation of T4 in ponies. Understanding physiological hormone concentrations and their natural variability across different equine species is essential for accurate interpretation of endocrine test results. Reliance on reference ranges established for adult and geriatric horses when evaluating young ponies or donkeys may increase the risk of overdiagnosis of endocrine disorders, including PPID, as well as hypothyroidism or hyperthyroidism.

The purpose of the present study was to assess resting ACTH and T4 values in healthy young equids, such as horses, donkeys, and ponies, during autumn and spring. We hypothesized that healthy young equids, especially ponies and donkeys, exhibit physiologically higher resting ACTH and T4 concentrations compared with healthy mature horses, and that these values are further affected by season and species differences.

## 2. Materials and Methods

The investigation involved 42 equids, which were divided into 3 groups: horses (control group), ponies, and donkeys. All animals were considered clinically healthy based on history, physical examination, and routine blood test results (complete blood count and serum biochemistry). They were in normal body condition (BCS 4–5/9) and maintained under conditions ensuring appropriate welfare. Preventive care followed standard guidelines and included deworming based on fecal examination, annual vaccination against equine influenza, tetanus vaccination every two years, and yearly dental examinations. Exclusion criteria included symptoms suggestive of clinical endocrine disease, illness, lameness or pharmacological treatment within the past six months, pregnancy or rearing of a foal under six months of age (pre-weaning), and changes in stabling or transportation within the preceding six months.

The horses were housed in stables with access to paddocks from 12 p.m. to 9 p.m. Ponies and donkeys were kept in a free-range system daily, with access to a large shelter providing protection from environmental conditions. Horses used the carousel daily and were trained for groundwork and racing, whereas ponies and donkeys were involved only in ground-based activities without carousel use or ridden exercise. In addition, the donkeys were trained for onotherapy. All animals had constant access to roughage (hay and straw) and mineral–vitamin supplements, while the horses received roughage adjusted to their individual nutritional requirements.

The ‘horse’ group included six Thoroughbred individuals aged 2.3 ± 0.8 years and of both sexes (four stallions, one gelding, and one mare). The ‘pony’ group consisted of 20 individuals aged 3.8 ± 1.18 years representing two breeds (six Falabella and fourteen Shetland ponies) and both sexes (eight stallions, one gelding, and eleven mares). The ‘donkey’ group included 16 individuals (*Equus asinus*) aged 4.2 ± 2.3 years of various breeds and both sexes (one stallion, four geldings, and eleven mares). Each group was maintained at a separate facility. However, all facilities were located within the same region of the country, characterized by similar climatic conditions, and were situated no more than 500 km apart. To evaluate seasonal variation in the investigated hormones, samples were collected in October 2020 (D1) and February 2021 (D2). Blood sampling was performed in the morning between 7:00 and 9:00 a.m., before any forced physical exercise. In horses, sampling was conducted at least one hour after the consumption of concentrate feed in order to standardize the postprandial endocrine status and minimize variability in ACTH and T4 concentrations [10]. Blood was collected from the external jugular vein using the Vacutainer system into tubes containing either a clot activator (for serum collection for T4 determination) or EDTA as an anticoagulant (for plasma collection for ACTH determination). After centrifugation, serum and plasma were separated, frozen at −20 °C, and transported to the laboratory for analysis [11]. For five individuals from the pony group, the second measurement could not be obtained due to relocation.

All analyses were performed in the commercial Polish Veterinary Laboratory VetLab. ACTH was determined via a two-step, chemiluminescent, solid-phase sequential immunometric assay (Immulite 2000, Siemens Medical Solutions USA, Inc., Malvern, PA, USA) in pmol/L. Two incubation cycles of 30 min each were performed. The assay achieved analytical precision of 5 pg/mL (1.1 pmol/L) and interassay precision (pg/mL) of 10.0%, 8.2%, 8.7%, 9.3%, and 6.1%. For comparison with existing literature, the units were converted using the following formula: ACTH [pmol/L] = ACTH [pg/mL] × 0.22. Total T4 was analyzed with a solid-phase, chemiluminescent immunoassay (Immulite 2000, Siemens Medical Solutions USA, Inc.) in units of µg/dL, which was originally designed for dogs but was validated by a commercial laboratory and used in the past for horses [12]. One incubation cycle was performed.

Statistical analyses were conducted using Microsoft Excel, STATISTICA software v.13.1 (StatSoft Inc., Tulsa, OK, USA), and Python v. 3.13.9 (SciPy library). Gender distribution across the study groups was compared using the Fisher–Freeman–Halton exact test for 3 × 2 tables (using Python). The distributions of age, ACTH, and T4 data were tested for normality with the Lilliefors test (using STATISTICA). Since the data deviated from normality, logarithmic transformation was applied to improve distribution characteristics. However, despite the transformation, the age data failed to meet the assumption of homogeneity of variance (Levene’s test, *p* < 0.05). Consequently, age was analyzed using the non-parametric Kruskal–Wallis ANOVA with post hoc multiple comparisons of mean ranks for all groups. For T4 and ACTH, a factorial ANOVA (factors: species and time) was performed, followed by an unequal N HSD post hoc test.

## 3. Results

No significant differences in gender proportions were observed between the groups (*p* = 0.0833). The Kruskal–Wallis test revealed a statistically significant difference in age between the studied groups (H (2) = 6.16, *p* = 0.0458). However, subsequent post hoc multiple comparisons did not identify specific significant differences between any two individual groups (*p* > 0.05 for all comparisons).

**Figure 1:**
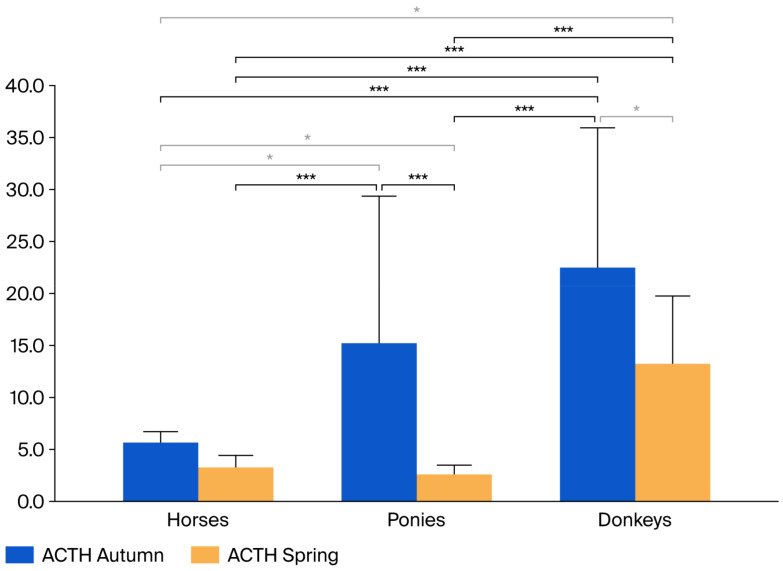
Mean concentrations of plasma ACTH (pmol/L) in the examined groups during autumn (2020) and spring (2021). Legend: Differences between groups are indicated by brackets. Black brackets with three asterisks (***) denote significance concentrations at *p* < 0.001, while gray brackets with a single asterisk (*) denote significance at *p* < 0.05.

The ACTH results for groups and periods are shown in Figure 1 and Table 1.

ACTH concentrations were significantly affected by species, time, and their interaction. Due to this significant interaction, post hoc analysis (Unequal N HSD) was conducted to identify specific differences between groups across all combinations of species and time points. The concentration of ACTH was significantly higher in autumn than in spring in all the animals (*p* = 0.00015). The concentration of ACTH was significantly higher in donkeys than in horses and ponies (*p* = 0001). ACTH concentrations measured in horses during spring were significantly lower than those observed in ponies in autumn (*p* < 0.001). There was a significant interaction effect between the group and time of sampling. The individual relationships of the ACTH values between the groups and periods are shown in Figure 1.

The T4 results for each group are shown in Table 2.

The factorial ANOVA revealed significant main effects for both species and time on T4 concentration. Since the interaction between groups and time of sampling was not significant, post hoc comparisons (Unequal N HSD) were performed separately to identify differences between species and between seasons. The concentration of T4 was significantly higher in autumn than in spring in all the animals (*p* = 0.049). The concentration of T4 was significantly higher in donkeys than in horses (*p* = 0.0046).

## 4. Discussion

This study provides an assessment of ACTH and T4 concentrations in 42 young, healthy equids divided into three groups—horses, ponies, and donkeys—conducted to identify interspecies differences as well as the impact of autumn and spring seasonality on these parameters. Our findings align with existing research indicating seasonal fluctuations in ACTH concentrations and higher ACTH concentrations in donkeys compared to horses and ponies, reinforcing the reliability and validity of the results. Furthermore, this study provides valuable species-specific diagnostic insights, underscoring the importance of establishing distinct ACTH reference ranges for young individuals across equine species, particularly in donkeys. These findings have important clinical implications for improving veterinary diagnostic accuracy.

ACTH is a hormone produced by the pituitary gland that stimulates the adrenal cortex to secrete cortisol. Analysis of the obtained ACTH results showed that, in the pony group, the mean ACTH concentration measured in autumn was higher than that observed in the horse group. This finding is consistent with the results reported by Bamford et al. (2023) and Vaughn et al. (2025), who also demonstrated that this difference occurs primarily during autumn, while values remain within normal ranges in spring [8,9]. Similarly, as in the available studies, ACTH concentrations in donkeys were significantly higher than those in horses during both periods studied [5,6,13]. This finding indicates the need to use a different reference standard for ponies and donkeys. In the paper by Gehlen et al. 2020, reference values for donkeys were adopted—19.5–143 pg/mL for the autumn period and 5.0–55.4 pg/mL for the spring period—corresponding to 4.29–31.46 pmol/L and 1.1–12.2 pmol/L, respectively [14]. The ACTH values observed in the present study exceeded the previously reported reference ranges for both autumn and spring. In addition, considerable individual variability in ACTH concentrations was observed within the donkey group, with some values exceeding the limits proposed by Gehlen et al. The higher ACTH values observed in the present study compared with those reported by Gehlen et al. may be explained by differences in age distribution, as the donkeys in that study ranged from 3 to 30 years of age, whereas the animals included in our study were considerably younger. The ACTH values obtained for horses in the present study are consistent with those reported in the literature (Table 3) and fall within the accepted reference ranges [4,15,16].

Interestingly, ACTH concentrations in young horses were similar in autumn and spring in the present study; however, this observation may be influenced by the relatively small sample size of the group. In young horses, the numerical increase in ACTH concentration in autumn was less pronounced compared to ponies and donkeys. Donkeys exhibited the greatest seasonal variation in ACTH concentrations and also had the highest ACTH concentrations in both seasons. Interspecies differences likely stem from inherent physiological traits of each species. Donkeys, for instance, are adapted to withstand harsher environmental conditions at different latitudes compared to horses and ponies. These differences may also relate to their behavioral responses to stress. Elevated concentrations of ACTH and cortisol have been documented in donkeys experiencing stressful conditions [5,17]. In ponies, breed-specific higher physiological ACTH responses to seasonal changes have been observed, which may be related to their adaptation to more severe environmental conditions compared to horses. For example, ponies are better able to tolerate poor diets and environmental stressors. This may suggest the presence of breeds or differences in pituitary functioning across species. Feeding prior to the ACTH measurement may increase ACTH concentrations [10]; however, in this study, only horses received concentrated feed, and their ACTH values remained lower than those of donkeys and ponies.

Age-related endocrine variation should also be considered when interpreting ACTH concentrations in equids. Previous studies have shown that hormonal profiles can differ with age, particularly for ACTH, which tends to increase in older animals due to age-related changes in pituitary function and the higher prevalence of PPID in geriatric horses. Consequently, younger animals may exhibit different baseline endocrine values compared with adult or aged individuals, which should be taken into account when comparing results across studies or populations [18,19]. In addition, potential pre-analytical factors affecting ACTH measurement should be taken into account. ACTH is a relatively unstable peptide hormone that undergoes rapid degradation if blood samples are not handled appropriately. Therefore, proper collection into EDTA tubes, immediate cooling, prompt centrifugation, and rapid plasma separation are recommended to preserve hormone stability. Inadequate sample handling or delayed processing may lead to artificially decreased ACTH values and contribute to variability between studies [19]. In the present study, all recommended precautions for ACTH sampling and handling were applied in order to minimize these potential effects.

A notable strength of this study is the novel demonstration of seasonal variation in T4 concentrations, marking the first report of statistically significant differences in serum T4 concentrations across seasons in equids. This phenomenon had not been previously documented in the literature. This is also the first study to demonstrate higher T4 concentrations in donkeys compared to horses. T4 is one of the hormones produced by the thyroid gland, and its activity may be indicative of organ function. Interpretation of T3 and T4 is challenging because blood thyroid hormone concentrations (either total or free) do not reflect the ability of the thyroid gland to respond during disease or to environmental changes. Changes in T4 can occur with, for example, sex, age, feeding, exercise, or transport [20,21,22]. Therefore, the use of a thyroid hormone panel including both total and free fractions of T4 and T3 has been recommended to reduce the risk of false diagnoses of hypothyroidism [23]. However, available evidence indicates that, in healthy individuals, total and free hormone concentrations are strongly correlated [20]. The present study confirmed significantly higher T4 concentrations in donkeys than in horses [20,21], as originally reported by Mendoza et al. (2013) [7]. The T4 value for ponies did not differ significantly from the accepted values for horses (1.0–4.1 ug/dL) [20,21]. The study confirmed seasonal variations in T4 concentrations across all groups. Previous research by Place et al. (2010) found that, in September, T4 concentrations were at the lower detection limit of the assay, whereas in March, significantly higher T4 values were observed compared to all other months, except for November and December [1]. However, the differences noted did not exceed the accepted reference limits for horses. None of the animals included in the study exceeded the literature reference limits for T4 in any of the collected samples. Similarly, in humans, seasonal fluctuations in T4 concentrations remain unclear, and research findings are often inconsistent. Some studies, especially in children, have reported higher T4 concentrations during colder months, which are attributed to an increased basal metabolic rate and heat production [24]. The T4 results and their seasonal variations may be influenced by the geographic location of the study group, as the magnitude of seasonal changes can vary depending on regional climate and environmental factors [25]. All animals included in the study originated from the same climatic region, as they were all kept in Poland. Studies have demonstrated that the type of diet ingested by horses can affect T4 concentrations. A diet lower in energy and protein may lead to increased T4 concentrations [26]. As donkeys and ponies have a poorer diet compared to horses, their T4 concentrations may have been influenced by these nutritional differences. The iodine concentrations in the diet were not measured in the study group; however, studies on ponies have indicated that dietary iodine does not significantly affect T4 concentrations [27].

The present study has several limitations, including a small sample size. The authors were keen to obtain homogeneous groups and to keep individuals within each group in separate breeding centers. This made it possible to exclude possible effects of housing and food on discrepancies within groups. Differences in activity level between the studied groups may also have influenced the endocrine results, as the horses were regularly exercised, whereas the donkeys and ponies were not used for training. Physical exercise has been shown to activate the hypothalamic–pituitary–adrenal axis and increase ACTH secretion, while also affecting thyroid hormone metabolism, including circulating T4 concentrations [28,29]. Nevertheless, despite their higher activity level, the horses in the present study exhibited lower ACTH and T4 concentrations than the donkeys. All animals underwent a routine clinical examination prior to sampling, including palpation of the cervical region (in the location of the thyroid gland), to exclude enlargement. However, ultrasonographic examination of this organ could not be performed due to the lack of owner consent for clipping and limited cooperation of some animals, particularly donkeys. The animals were kept in Poland to exclude the possible influence of geographical changes on the results obtained, as confirmed in the studies by McFarlane et al. 2011 and Secombe et al. 2017 [30,31]. Both of the cited studies involved distances of more than 10,000 km, whereas in the work presented here, it would have been a distance of approximately 500 km. Another limitation of the study was the inability to assess T3, fT3, and fT4 concentrations due to laboratory constraints, including the lack of facilities in Poland performing these analyses in equids, as well as financial limitations. Future investigations incorporating these parameters and involving larger populations could provide a more comprehensive evaluation of thyroid hormone profiles, species-related differences in equids, and the establishment of robust reference values. Expanding the research to include young equids with endocrine disorders could help define physiological threshold values; however, this may be challenging due to the relative rarity of such conditions in this age group. The present study focused on young, clinically healthy equids to avoid confounding physiological variation with pathological changes in clinical practice.

## 5. Conclusions

Both ACTH and T4 showed significant seasonal variation, with consistently higher concentrations recorded in autumn than in spring across all species. The study demonstrates that young donkeys exhibit significantly higher ACTH concentrations than horses or ponies; moreover, they were found to have notably higher T4 concentrations compared to horses. The results indicate that higher reference values should be applied when testing ACTH and T4 concentrations in donkeys, as well as ACTH concentrations in ponies.

## Figures and Tables

**Table 1:** Mean plasma ACTH concentrations (pmol/L) in the studied animal groups during autumn and spring are presented, with minimum and maximum values in parentheses.

Group of Animals	Autumn	Spring
Horses	5.7 ± 1.1 (3.7–8.5)	3.4 ± 1.0 (1.9–5.7)
Donkeys	22.5 ± 13.4 (7.1–53.9)	13.4 ± 6.4 (5.4–27.8)
Ponies	15.2 ± 14.2 (4.1–37.8)	2.7 ± 0.8 (1.5–4.4)

**Table 2:** Mean serum T4 concentrations (ug/dL) in the studied animal groups during autumn and spring are presented, with minimum and maximum values in parentheses.

Group of Animals	Autumn	Spring
Horses	1.7 ± 0.8 (0.66–3.70)	0.9 ± 0.3 (0.42–1.58)
Donkeys	2.9 ± 1.6 (0.68–8.52)	2.5 ± 1.4 (0.61–7.45)
Ponies	2.2 ± 0.8 (0.87–4.64)	1.8 ± 0.7 (0.69–3.84)

**Table 3:** Collected average ACTH concentrations in equines, as reported across previous research studies.

References	Autumn Value (Sept.–Oct.)	Spring Value (Dec.–Jun.)
EEG Recommendation 2023 [4]	<6.6 pmol/L	<3.3 pmol/L
EEG 2023 Equivocal [4]	6.6–19.8 pmol/L	3.3–8.8 pmol/L
Copas and Durham, 2012 [15]	10.35 pmol/L	6.44 pmol/L
Reference standards for horses approved by the laboratory performing the current analysis, according to Gimplinger, Fey 2012 [16]	11.30 pmol/L	7.90 pmol/L

## Data Availability

The data that support the findings of this study are available from the corresponding author upon reasonable request.

## Abbreviations

The following abbreviations are used in this manuscript:

ACTH	adrenocorticotropic hormone
BCS	body condition score
PPID	pituitary pars intermedia dysfunction
T4	thyroxine
